# Development of a fall-risk assessment profile for community-dwelling older adults by using the National Health Interview Survey in Taiwan

**DOI:** 10.1186/s12889-020-8286-8

**Published:** 2020-02-14

**Authors:** Ping-Ling Chen, Hsiao-Yu Lin, Jiann Ruey Ong, Hon-Ping Ma

**Affiliations:** 1 Graduate Institute of Injury Prevention and Control, College of Public Health, Taipei Medical University, Taipei, Taiwan; 2 Department of Urology, Taipei Medical University Hospital, Taipei, Taiwan; 3 Emergency Department, Shuang-Ho Hospital, Taipei Medical University, Taipei, Taiwan; 4 Emergency Department, School of Medicine, Taipei Medical University, Taipei, Taiwan

**Keywords:** Older, Fall, National Health Interview Survey, Risk assessment profile

## Abstract

**Background:**

Falls represent a global health issue among older adults and cause a considerable burden on medical systems. In this study, a fall-risk assessment profile was developed for community-dwelling older adults.

**Method:**

The data of survey participants aged > 65 years were obtained from three rounds (2005, 2009, and 2013) of the National Health Interview Survey in Taiwan. In total, 8356 older participants were included in this study. Logistic regression analyses were used to determine potential predictors associated with falls. The regression coefficients of the predictors in the final model were translated into scores (by multiplying by 5) and then summed to obtain a total risk-score for falls. A receiver operating characteristic (ROC) curve was used to evaluate the discriminative performance of the risk assessment profile.

**Result:**

Self-reported falls within 1 year accounted for 19.1% of the total falls. The predictors that were included in the risk profile according to the logistic regression analysis results were as follows: female sex (adjusted odds ratio = 1.57; risk-score = 2), living alone (adjusted odds ratio = 1.56; risk-score = 2), urinary incontinence (adjusted odds ratio = 1.36; risk-score = 2), perceived unhealthiness (adjusted odds ratio = 1.32; risk-score = 1), perceived pain (adjusted odds ratio = 1.51; risk-score = 2), hospital admission in the past year (adjusted odds ratio = 2.42; risk-score = 4), low activity of daily living (ADL) scores (adjusted odds ratio = 1.29; risk-score = 1), and low mobility function scores (adjusted odds ratio = 1.68; risk-score = 3). At a total risk-score cutoff point of 6 (range 0–17), the model predicted falls with a sensitivity and specificity of 75.16 and 52.75%, respectively (area under the ROC curve = 0.70).

**Conclusion:**

The fall-risk assessment profile comprising eight predictors—female sex, living alone, incontinence, perceived unhealthiness, perceived pain, hospital admission in the past year, low ADL scores, and low mobility function scores—may serve as an assessment tool for identification of older adults with a high risk of falling, and assessment results can be used to facilitate community-based intervention.

## Background

Falls represent a major cause of disability and death, particularly in the older population, and contribute to serious public health problems worldwide [1]. Age is a well-known risk factor for falls. Age-related injuries resulting from falls are expected to increase considerably due to an increase in the proportion of older-aged individuals in the overall population. The incidence of falls varies worldwide [2–4]. Approximately one quarter of adults aged > 60 year-old experience at least one fall, and one out of five falls results in serious injury associated with a substantial burden not only on the older adults but also on their families and society; moreover, fall-related injuries are associated with high medical expenses and health care demands [5–7]. The consequences of falls are serious in the older adult population [8].

Falls in older adults can be prevented. A history of falls is associated with a high risk of recurrent falling [9, 10]. The prevention of falls has become a crucial research area because of the severe potential consequences of falling. Identification of potential factors associated with falls may facilitate development of an effective fall-prevention program. Several studies have reported interventions that reduced the risk of falling [11–13]. Over many years, epidemiological data have been collected to identify fall-related risk factors, and fall-prevention programs have been proposed and evaluated [5, 14, 15]. In a report published by the World Health Organization in 2008, the main risk factors for falls were categorized as follows: biological risk factors (e.g., age), behavioral factors (e.g., alcohol use), environmental factors (e.g., home hazards), and socioeconomic factors (e.g., income) [1]. However, the risk profiles of falls in older adults may vary across countries and cultures [16]. A comprehensive study for the development of a risk profile to predict recurrent falls among older individuals was proposed in 2006 [17], but this risk profile was for community-dwelling older adults in the Netherlands. An appropriate risk assessment instrument for Asian older adults needs to be developed.

In this study, we developed a risk assessment profile for falls in older adults by using a national database, the National Health Interview Survey (NHIS) in Taiwan. The predictors of the risk of falling in older patients were investigated, and a total risk score was calculated to identify older adults at a high risk of falling.

## Methods

### Data source

The NHIS is a large-scale, cross-sectional, and face-to-face survey that is conducted once every 4 years by the Health Promotion Administration, National Health Research Institutes, Food and Drug Administration, and Ministry of Health and Welfare of Taiwan. The participants in the NHIS were sampled using a multistage probability proportional to sample size technique. Survey questionnaires were prepared for three age groups, namely, ≥65 years, 12–64 years, and ≤ 11 years. In this study, the participants aged ≥65 years were obtained from the NHIS 2005, 2009, and 2013 databases, respectively. This study was approved by the Taipei Medical University-Joint Institution Review Board (TMU-JIRB N201612015), and the data were provided by the Health and Welfare Data Science Center.

The participants recalled their fall experiences in the past year, including falls caused by slipping, walking, dizziness, sitting, standing, or lying down. Several aspects of physical and social functioning were also assessed in the NHIS, and the potential predictors of the risk of falling were classified into four categories: demographic characteristics, health status, activity or mobility, and life style. In total, 34 potential predictors listed in the NHIS database were selected for developing the risk profile of falling for the older participants.

The demographic characteristics included age (> 75 years or ≤ 75 years), sex, living in a highly urbanized area (the first of seven clusters defined by Liu et al. [18], living alone, marital status, working status, and income. The monthly income of the entire family was categorized as < 30,000 New Taiwan dollars (NTD) (equal to 1000 USD) or ≥ 30,000 NTD. The participants were also asked to specify their highest completed educational level, and a high education level was defined as ≥10 years of education. The following 19 variables were identified under the health status category: diabetes, hyperlipidemia, asthma, cardiovascular disease, osteoporosis, psychological disease, epilepsy, Parkinson’s disease, dementia, osteoarthritis, urinary incontinence, hypertension, visual impairment, hearing impairment, paralysis of limbs, poor self-reported health status, pain (including pain in the arms, hips, knees, chest, and back), hospital admission in the past year, and body mass index (BMI). BMI was calculated using body weight and height, and participants with BMI ≤24 kg/m^2^ and those with BMI > 24 kg/m^2^ were compared.

A poor mobility status was defined as a score ≥ 1 on one or more mobility tests. The functional limitations of the participants were assessed based on activity of daily living (ADL) and instrumental activity of daily living (IADL) scores, including scores for core daily personal self-care tasks (e.g., eating). Low ADL and IADL scores reflected difficulty performing more than two activities. Four life style variables, namely regular exercise, alcohol use, current smoking status, and betel nut chewing, were evaluated.

### Statistical analysis

Fall-related risk factors were identified using a multistep process. First, prevalence, percentage of missing values, and univariate logistic regression were calculated for each potential variable. The Spearman correlations between the variables were also calculated. Variables were excluded if their prevalence was less than 10%, the number of missing values exceeded 10%, or *p* > 0.2 in univariate logistic regression. Additionally, if two variables were highly correlated (Spearman correlation 0.4), the variable that was more easily measured was retained. After excluding the non-eligible variables, multivariable logistic regression and backward elimination with a stay-significance level of 0.2 were applied to identify the potential predictors to be included in the risk profiles of falling in the older adults. The modified falling risk profile was investigated and the weight (score) of each predictor was defined as the regression coefficient multiplied by 5 and rounded off to the nearest integer. A total risk-score was calculated for each participant. The evaluation values used in this study were negative predictive value (NPV), positive predictive value (PPV), sensitivity, and specificity. PPV indicated the probability of falling among the participants who were in the high-risk group, and NPV indicated the probability of not falling among the participants who were in the low-risk group. Sensitivity represented the probability of correctly identifying the older participants who experienced falls (fallers) as having at least one fall, and specificity was the probability of correctly identifying the older patients who did not experience falls (nonfallers). The diagnostic value was evaluated based on the receiver operator characteristic (ROC) curve, and the optimal cutoff point value was defined as the point corresponding with the maximum summed sensitivity and specificity. The analyses were performed using SAS software, Version 9.4 in the SAS system for Windows.

## Results

### Demographic characteristics

The baseline characteristics of the participants are listed in Table 1. From three survey rounds, 8356 participants aged ≥65 years were included in this study, and of these, 1589 (19%) had at least one fall event in the previous year. Overall, 22.1% female and 15.8% male participants reported at least one fall in the previous year. The percentage of the participants who experienced at least one fall in the past year was higher among those aged > 75 years than among those aged 65–75 years. In addition, participants with chronic diseases, such as diabetes, hyperglycemia, asthma, and cardiovascular disease, had a higher percentage of experiencing at least one fall in the past year than did those who did not have chronic diseases. The participants with low ADL scores or activity functions exhibited a > 2-times higher risk of falling than the participants with high ADL scores or activity functions. Moreover, the participants who did not smoke, or consume alcohol had a higher percentage of falls in the past year than those with these habits. However, the participants who chewed betel nut had a higher percentage of falls in the past year than those without these habits.

**Table 1:** Prevalence, univariate odds ratios (ORs), and 95% confidence intervals (CI) for potential predictors of falling

Variable	Number		Missing rate(%)	Prevalence	OR	95% CI of OR (*p*-value)
	faller	Not faller				
Total	1598	6758		19.1		
Gender			0			
Male	617	3295		15.8	Reference	–
Female	981	3463		22.1	1.51^*^	1.35–1.69(< 0.01)
Age			0			
65–75 years old	792	3917		16.8	Reference	–
≥ 75 years old	806	2841		22.1	1.40^*^	1.26–1.57(< 0.01)
Area			0.28			
Non-urbanized	1255	5241		19.3	Reference	–
Urbanized	337	1500		18.3	0.94	0.82–1.07 (0.34)
Education			0.78			
Not high	1434	5662		20.2	Reference	–
High	154	1041		12.9	0.58^*^	0.49–0.70(< 0.01)
Living alone			0			
No	1374	6019		18.6	Reference	–
Yes	224	739		23.3	1.33^*^	1.13–1.56(< 0.01)
Marital status			0			
Married	886	4320		17.0	Reference	–
Single or others	712	2438		22.6	1.43^*^	1.28–1.59(< 0.01)
Working status			0			
Working	174	1069		14.0	Reference	–
Retired or others	1424	5689		20.0	1.53^*^	1.29–1.81(< 0.01)
Income			4.61			
≥ 30,000	732	3307		18.1	Reference	–
< 30,000	799	3133		20.3	1.15^*^	1.03–1.29 (0.01)
Diabetes			0.14			
No	1203	5462		18.0	Reference	–
Yes	391	1288		23.3	1.38^*^	1.21–1.57(< 0.01)
Hyperlipidemia			1.40			
No	1148	5095		18.4	Reference	–
Yes	415	1581		20.8	1.17^*^	1.03–1.32 (0.02)
Asthma			0			
No	1490	6396		18.9	Reference	–
Yes	108	362		23.0	1.28^*^	1.03–1.60 (0.03)
Cardiovascular disease			0			
No	1228	5633		17.9	Reference	–
Yes	370	1125		24.7	1.51^*^	1.32–1.72(< 0.01)
Osteoporosis			0.22			
No	1108	5298		17.3	Reference	–
Yes	486	1446		25.2	1.61^*^	1.42–1.82(< 0.01)
Psycho disease			0			
No	1542	6582		19.0	Reference	–
Yes	56	176		24.1	1.36^*^	1.002–1.85 (0.04)
Epilepsy			0			
No	1589	6744		19.1	Reference	–
Yes	9	14		39.1	2.73^*^	1.18–6.31 (0.02)
Parkinson’s disease			0.13			
No	1539	6644		18.8	Reference	–
Yes	52	110		32.1	2.04^*^	1.46–2.85(< 0.01)
Dementia			0			
No^a^	1482	6550		18.5	Reference	–
Yes	116	208		35.8	2.46^*^	1.95–3.11(< 0.01)
Osteoarthritis			0			
No^a^	1307	5921		18.1	Reference	–
Yes	291	837		25.8	1.58^*^	1.36–1.83(< 0.01)
Urinary incontinence			0.34			
No	1068	5321		16.7	Reference	–
Yes	523	1416		27.0	1.84^*^	1.63–2.08(< 0.01)
Hypertension			0.12			
No	739	3561		17.2	Reference	–
Yes	855	3191		21.1	1.29^*^	1.16–1.44(< 0.01)
Visual impairment			0.11			
No	277	1288		17.7	Reference	–
Yes	1321	5461		19.5	1.13	0.98–1.30 (0.11)
Hearing impairment			0.04			
No	1192	5486		17.8	Reference	–
Yes	406	1269		24.2	1.47^*^	1.30–1.67(< 0.01)
Paralysis of limbs			0.69			
No	1381	6183		18.3	Reference	–
Yes	203	531		27.7	1.71^*^	1.44–2.03(< 0.01)
Self-report health status			9.16			
Health	488	3248		13.1	Reference	–
poor	896	2959		23.2	2.02^*^	1.79–2.28(< 0.01)
Pain			2.42			
No	519	3386		13.3	Reference	–
Yes	1053	3196		24.8	2.15^*^	1.92–2.41(< 0.01)
Hospital visit in the past year			0			
No	940	5337		15.0	Reference	–
Yes	658	1421		31.6	2.63^*^	2.34–2.95(< 0.01)
BMI			9.72			
> 24	696	2848		19.6	Reference	–
≤ 24	724	3276		18.1	0.90	0.81–1.02 (0.09)
ADLs			0.08			
< 3 activities	1299	6135		17.5	Reference	–
≥ 3 activities	299	616		32.7	2.29^*^	1.97–2.67(< 0.01)
IADLs			0.3			
Good	833	4767		14.9	Reference	–
Poor	761	1970		27.9	2.21^*^	1.98–2.47(< 0.01)
Mobility			0.35			
Good	433	3414		11.3	Reference	–
Poor	1161	3319		25.9	2.76^*^	2.45–3.11(< 0.01)
Regular exercise			3.47			
No	1445	6050		19.3	Reference	–
Yes	82	489		14.4	0.70^*^	0.55–0.89(< 0.01)
Alcohol consumer			0.37			
No	1434	5767		19.9	Reference	–
Yes	157	967		14.0	0.65^*^	0.55–0.78(< 0.01)
Current smoker			0			
No	1439	5863		19.7	Reference	–
Yes	159	895		15.1	0.72^*^	0.61–0.86(< 0.01)
Betel nut chewing			0			
No	1536	6536		19.0	Reference	–
Yes	62	222		21.8	1.19	0.89–1.58 (0.24)

The odd of falling in the female participants was 1.51 times higher than that in the male participants (95% confidence interval [CI], 1.35–1.69). The odd of falling in the participants aged > 75 years was 1.40 times higher than that in the participants aged 65–75 years (95% CI, 1.26–1.57).

### Potential predictor selection

**Fig. 1:**
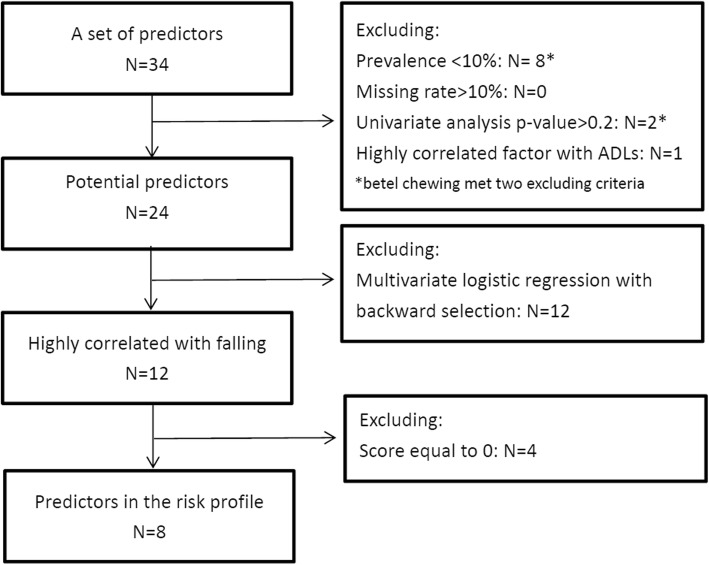
The study flow chart. N: number of risk factor

The flow chart illustrating the selection of the potential factors is presented in Fig. 1. The prevalence of the following eight factors was less than 10%: asthma, psychological disease, epilepsy, Parkinson’s disease, dementia, paralysis of limbs, regular exercise, and betel nut chewing. The percentages of missing values for each factor are indicated in the sixth column of Table 1. The proportion did not exceed 10% for any factor. In addition, the odds ratios and corresponding *p*-values from the univariate analysis for each potential factor are denoted in the fifth column of Table 1. Two factors, namely living in urban areas and betel nut chewing, were nonsignificant (*p* > 0.2). Moreover, the ADL score, which was relatively easy to measure, was significant and highly correlated with the IADL score. Therefore, the ADL score was retained as a factor, and the IADL score was excluded. After applying the exclusion criteria, 24 potential predictors remained in the risk profile of falling.

All 24 eligible factors were assessed in the multivariable regression model by using backward elimination, and the 12 factors selected for the final risk profile were age, sex, living alone, education, work status, diabetes, urinary incontinence, self-reported health status, pain, hospital admission in past year, ADL score, and mobility. The results are displayed in Table 2, and four of the factors, namely age, education, working status, and diabetes prevalence, exhibited a score of 0 and *p* > 0.05. The older participants who had been admitted to the hospital in the past year exhibited a high risk-score (4) in the risk profile of falling. The participants with low mobility scores exhibited a risk-score of 3. Some of the participants who were women, were living alone, had urinary incontinence, or experienced pain exhibited risk-scores of 2. The older participants with poor perceived health status or low ADL score, exhibited risk-scores of 1.

**Table 2:** Risk profile of falling among older

Predictors	Regression coefficient	Odds Ratio	P-value	Score
Constant	−2.73			
Age (≥75)	0.08	1.08	0.28	0
Female	0.45	1.57^*^	0.02	2
Live alone	0.45	1.56^*^	< 0.01	2
High education	−0.09	0.91	0.59	0
Retired	−0.03	0.98	0.86	0
diabetes	0.003	1.00	0.99	0
Involuntary loss of urine	0.30	1.36^*^	< 0.01	2
Self-perceived Health (poor)	0.28	1.32^*^	0.02	1
Pain	0.41	1.51^*^	< 0.01	2
Hospital admission in the past year	0.88	2.42^*^	< 0.01	4
ADLs (poor)	0.25	1.29^*^	0.03	1
Mobility (poor)	0.52	1.68^*^	< 0.01	3

^*^: p-value < 0.05

**Fig. 2:**
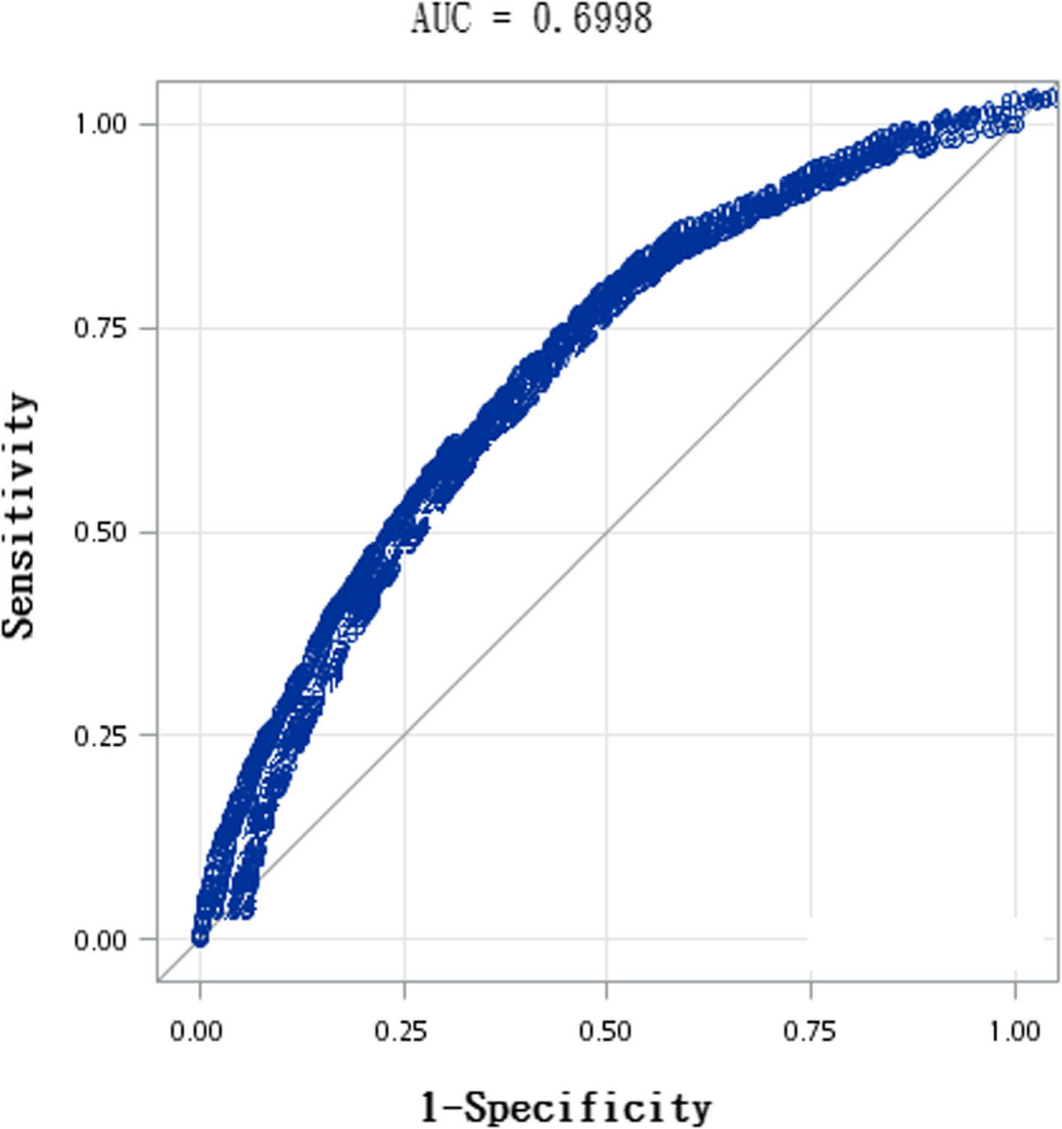
ROC curve

The ROC curve of the risk of falling profile in the older participants is shown in Fig. 2, and the area under the ROC curve (AUROC) is 0.70. The NPV, PPV, sensitivity, and specificity assessed for different cutoff values in the total risk-score are shown in Table 3. The NPV, PPV, sensitivity, and specificity at a cutoff point of 1 were 20.42, 93.46, 96.81, and 10.79%, respectively. The sensitivity was moderate and the specificity was low at a relatively low cutoff. When the cutoff score increased, the sensitivity decreased and the specificity increased. The maximum summation of sensitivity and specificity was reached at a score of 6 (scores of 0–5 versus ≥6), and its corresponding PPV, NPV, sensitivity and specificity were 27.33, 89.98, 75.16, and 52.75%. At a cutoff score of 6, 24.84% of the fallers were not included in the high-risk group, and 47.25% of the nonfallers were included in the high-risk group. With a cutoff score of 11 (score of 0–10 versus ≥11), the sensitivity was low (26.2%) and the specificity was high (90%).

**Table 3:** Sensitivity and specificity at different cut-off points in the total risk score

Cut-off in the total risk score	A(n)	B(n)	C(n)	D(n)	Sensitivity (%)	Specificity (%)	Σ%	PV+(%)	PV-(%)
0 vs. ≥1	1547	6029	51	729	96.8	10.8	107	20.4	93.5
0–1 vs. ≥2	1522	5773	76	985	95.2	14.6	110	20.9	92.8
0–2 vs. ≥3	1450	4923	148	1835	90.7	27.2	118	22.8	92.5
0–3 vs. ≥4	1401	4367	197	2391	87.7	35.4	123	24.3	92.4
0–4 vs. ≥5	1315	3808	283	2950	82.3	43.7	126	25.7	91.2
0–5 vs. ≥6	1201	3193	397	3565	75.2	52.8	128^a^	27.3	90.0
0–6 vs. ≥7	1063	2621	535	4137	66.5	61.2	128^a^	28.9	88.5
0–7 vs. ≥8	911	2091	687	4667	57.0	69.1	126	30.3	87.2
0–8 vs. ≥9	720	1427	878	5331	45.1	78.9	124	33.5	85.9
0–9 vs. ≥10	604	1099	994	5659	37.8	83.7	122	35.5	85.1
0–10 vs. ≥11	419	641	1179	6117	26.2	90.5	117	39.5	83.8
0–11 vs. ≥12	335	470	1263	6288	21.0	93.0	114	41.6	83.3
0–12 vs. ≥13	189	208	1409	6550	11.8	96.9	109	47.6	82.3
0–13 vs. ≥14	136	142	1462	6616	8.5	97.9	106	48.9	81.9
0–14 vs. ≥15	43	39	1555	6719	2.7	99.4	102	52.4	81.2
0–15 vs. ≥16	12	10	1586	6748	0.8	99.9	101	54.5	81.0
0–16 vs. ≥17	4	3	1594	6755	0.3	100.0	100	57.1	80.9

A, number of participants who were assigned to the high-risk group and who were fallers; B, number of participants who were assigned to the high risk group and who were not fallers; C, number of participants who were assigned to the low-risk group and who were fallers; D, number of participants who were assigned to the low-risk group and who were not fallers; Σ Sum of sensitivity (A/(A + C))and specificity (D/(B + D)); PV+: Positive predictive value (A/(A + B)); PV- Negative predictive value (D/(C + D))

^a^Maximal sum of sensitivity and specificity

## Discussion

The series of nationwide surveys revealed that the risk of falling in the older participants with at least one fall could be predicted using a risk profile based on eight predictors. The participants who were women, lived alone, experienced urinary incontinence, self-reported poor health status, experienced pain, had undergone hospital admission in the past year, had low ADL scores, or had low mobility scores exhibited a high risk of falling. The AUROC was 0.7 for the proposed risk assessment instrument. The corresponding sensitivity and specificity varied with cutoff scores. At a cutoff score of 6, the summation of sensitivity (75.16%) and specificity (52.75%) was maximal.

Our results revealed that the risk of falling differed in the male and female participants. The reason for the higher risk in the female participants than in the male participants may have been the loss of bone mineral density associated with menopause [19]. However, a previous study showed that after the first occurrence of a fall, sex did not significantly affect the risk of recurrent falls; hence, sex was not included in the risk profile for recurrent falls [17]. Living alone has been identified as a significant risk factor; several studies have shown that older adults living alone are > 2 times more likely to experience a fall than those who do not live alone [20–22]*.* In this study, living alone was a significant risk factor for falling. One health risk, urinary incontinence, was observed in more than one-third of older adults who lived in communities, had long stay durations in institutions, or had undergone hospitalization. Our study indicated that urinary incontinence was a risk factor for falls, and several cross-sectional epidemiological studies have shown a significant association between the occurrence of falls and urinary incontinence [23, 24]. Chronic pain is strongly associated with falls, and pain is common in older adults (up to 76%) [25, 26]. In our study, general pain status (for all pain locations) was assessed, and it was a risk factor for falls with a score of 2 among the older participants. Pain has been reported to be a risk factor for recurrent falling; therefore, an older patient who experiences pain requires close attention [27].

In our study, low ADL scores and low mobility were associated with high risk of falling. Similar results have been previously reported [28, 29]. These results show that facilitating the maintenance of healthy ADLs in older adults is necessary, but some mobility behaviors may be associated with the risk of falling [30]. Home safety modifications have been suggested to reduce the risk of falling during the performance of ADLs [31, 32]. Some studies have investigated the risk of falling in hospitalized patients [33, 34]. Researchers have also examined hospital admissions that resulted in falls [35]. In our study, the hospital admission was a significant risk factor for falling in the proposed risk assessment instrument.

All the significant risk factors from our study have been presented separately in previous studies. In this study, these risk factors were evaluated simultaneously. A modified falling risk assessment instrument was proposed; health providers can use this instrument to easily assess older adults’ risk of falling. In this study, the total risk-score was calculated, facilitating identification of patients with high risk of falling. In practice, health providers can refer high-risk patients to relevant intervention programs to prevent falls.

This study exhibited several strengths. The risk profile was developed based on a large sample, which was selected by an experienced survey group through a standard interview process. The population-based sample used in this study was representative of the population of older adults in Taiwan.

A limitation in this study was that some data, such as physical activity volume, were self-reported. This may have reduced the accuracy of data and thus resulted in recall bias. The fall experience was recalled retrospectively, and this may result in recall bias. Another limitation was that impact and severity of falls were not evaluated in this study. Moreover, our predicting model did not include a history of falls, which is known as a strong predictors of falls.

## Conclusion

Based on the data obtained from three rounds of the NHIS in Taiwan, an instrument for the assessment of the risk of falling in older adults (in Taiwan) was developed. A friendly tool was proposed to identify older adults with a high risk of falls. Identification of these individuals may reduce the number of injuries and fractures resulting from falls.

## Data Availability

The data that support the findings of this study are available from Health Promotion Administration, National Health Research Institutes, Food and Drug Administration, and Ministry of Health and Welfare of Taiwan but restrictions apply to the availability of these data, which were used under license for the current study, and so are not publicly available. Data are however available with permission of Health Promotion Administration, National Health Research Institutes, Food and Drug Administration, and Ministry of Health and Welfare of Taiwan.

## Abbreviations

ADL: Activity of daily living
AUROC: Area under the ROC curve
BMI: Body mass index
CI: Confidence interval
IADL: Instrumental activity of daily living
NHIS: National Health Interview Survey
NPV: Negative predictive value
PPV: Positive predictive value
ROC: Receiver operating characteristic
